# Orthognathic surgery and orthodontics associated with orofacial harmonization: Case report

**DOI:** 10.1016/j.ijscr.2021.106013

**Published:** 2021-05-26

**Authors:** Alessandra Kuhn Dall'Magro, Letícia Copatti Dogenski, Eduardo Dall'Magro, Nicoly Schmidt Figur, Micheline Sandini Trentin, João Paulo De Carli

**Affiliations:** a Department of Bucomaxillofacial Surgery, São Vicente de Paulo Hospital, Passo Fundo, Brazil; b Post-graduation Program in Oral Diagnosis, Faculty of Dentistry, Universidade Federal de Santa Catarina, Florianópolis, Brazil; c Department of Restorative Dentistry, Faculty of Dentistry, Universidade de Passo Fundo, Passo Fundo, Brazil; d Faculty of Dentistry, Universidade de Passo Fundo, Passo Fundo, Brazil; e Department of Periodontics, Faculty of Dentistry, Universidade de Passo Fundo, Passo Fundo, Brazil; f Departament of Oral Medicine and Prosthodontics, Faculty of Dentistry, Universidade de Passo Fundo, Passo Fundo, Brazil

**Keywords:** Orthognathic surgery, Orofacial harmonization, Orofacial esthetics, Case report

## Abstract

**Introduction and importance:**

Orthognathic surgery aims to restore the functional and esthetic standards of the face and non-surgical or minimally invasive procedures have been optimizing the results of facial orthosurgical treatments. This case report aimed to show the use of minimally invasive techniques that, associated with orthognathic surgery, represent a trend in current oral and maxillofacial rehabilitation.

**Case presentation:**

A female patient, 28 years old, white, sought care from the Dentistry team of the São Vicente de Paulo Hospital, in Passo Fundo, Brazil, complaining of mandibular prognathism, anteroposterior maxillary deficiency, dental crowding, malocclusion, functional and esthetic changes that negatively affected her psychosocial interactions and stomatognathic function. The treatment involved orthodontics, orthognathic surgery, and orofacial harmonization with dermal fillers.

**Clinical discussion:**

The multidisciplinarity among the specialties of Oral and Maxillofacial Surgery and Traumatology, Orthodontics, and Orofacial Harmonization add and contribute to the process of planning and implementing the treatment proposed, as well as the prognosis toward patient satisfaction.

**Conclusion:**

The surgical procedure associated with minimally invasive facial harmonization not only corrected the functional complaint of the patient but also played an important role in improving facial harmony, contributing significantly to self-esteem.

## Highlights

• Minimally invasive facial harmonization refines orthognathic surgery techniques.
• The integration of several disciplines helps to improve facial harmony.
• Injectable fillers represent important allies in orthosurgical treatments.

## 1 Introduction and importance

Many patients seek dental and maxillofacial surgery not only to improve function but also for esthetic improvements in the smile or face. Therefore, orthognathic surgery has a potential impact on the quality of life, psychosocial well-being, facial esthetics, and oral function [1]. It is performed to correct dentoskeletal discrepancies and seek harmony between upper and lower jaws, improving occlusal function. Furthermore, it offers the benefits of improving the self-esteem, satisfaction, self-confidence, social functioning, and interpersonal relationships of patients [2].

The management of most orthognathic surgery cases includes preoperative orthodontics to decompose the dentition, followed by surgery and postoperative orthodontics [1]. After removing the dental compensations before surgery, this technique allows the optimal surgical repositioning of the mandible. In the search for satisfactory results in both function and post-surgical orofacial esthetics, the facial contouring with injectable fillers such as hyaluronic acid (HA) may serve as a complementary finishing touch to obtain volume correction, improving the shape and facial asymmetries. The main options for using these substances include eyebrow positioning, nasal tip elevation, jawline modeling, chin shape and definition, and lip eversion [3].

Thus, orofacial harmonization may integrate treatment plans to combine function, esthetics, and dental health, providing balance to a face that needs symmetry adjustments and balance between facial thirds. When carefully applied, injectable fillers provide an effective solution to several esthetic issues [4] and may represent important allies in orthosurgical treatments. Therefore, this study aims to show, through a case report, the multidisciplinary use of orthognathic surgery combined with orthodontics and minimally invasive techniques that, in association, present a trend in current oral and maxillofacial rehabilitation.

## 2 Case presentation

Written informed consent was obtained from the patient for publication of this case report and accompanying images. A copy of the written consent is available for review by the Editor-in-Chief of this journal on request. All applicable rules regarding the ethics of experimentation and research integrity were followed. This work has been reported in line with the SCARE 2020 [5] criteria.

Female patient, 28 years old, white, with good health, no history of drug use or any relevant family or genetic disease information, sought assistance from the Dentistry team at the São Vicente de Paulo Hospital, in Passo Fundo, Rio Grande do Sul, Brazil, for orofacial rehabilitation, complaining of mandibular prognathism, anteroposterior maxillary deficiency, dental crowding, malocclusion, and functional and esthetic changes (Angle Class III). The patient reported being disturbed by the lack of harmony on her face and the occlusal and functional problems developed over time due to the skeletal discrepancy that negatively affected her psychosocial interactions.

**Fig. 1:**
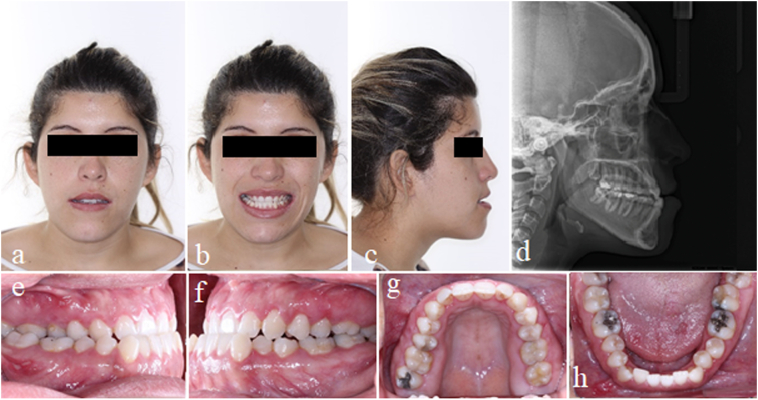
Initial frontal photos at rest (1a), smiling (1b), profile (1c), and profile teleradiography (1d). Initial intraoral photos of the right side (1e), left side (1f), upper arch (1g), and lower arch (1h).

**Fig. 2:**
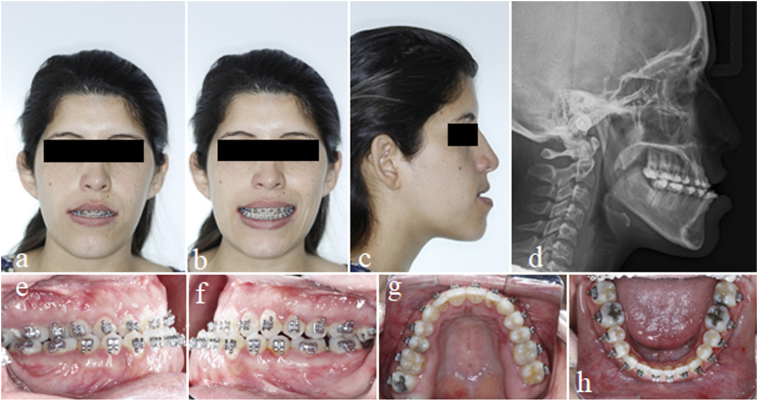
Photos after orthodontic preparation - leveling, dental alignment, and coordination of upper and lower arches in frontal view at rest (2a), smiling (2b), profile (2c), and profile teleradiography (2d). Intraoral photos after orthodontic preparation on the right side (2e), left side (2f), upper arch (2g), and lower arch (2h).

After an interdisciplinary evaluation, the treatment was planned according to the following sequence:

Phase 1 - Photos and initial documentation, followed by orthosurgical preparation with leveling, alignment, and coordination of the dental arches (Figs. 1 and 2).

**Fig. 3:**
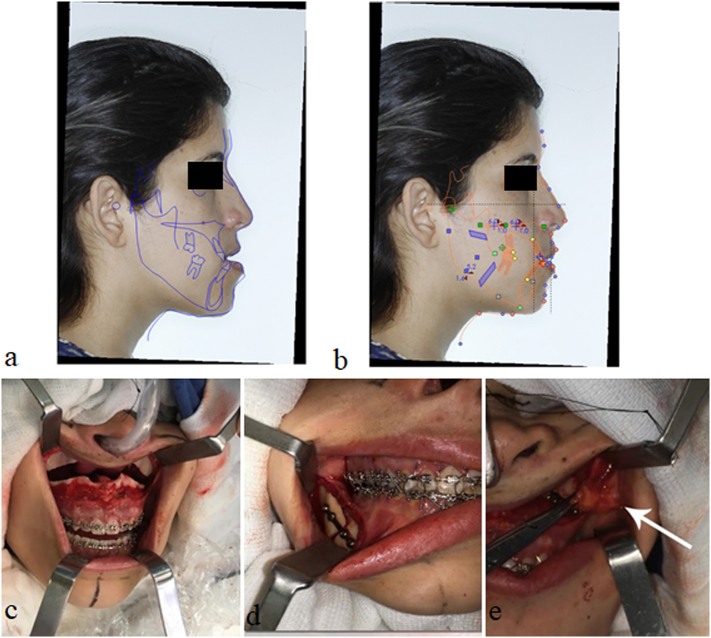
Virtual planning for predictive analysis through preoperative cephalometric evaluation of the Nemoceph™ software. An initial cephalometric analysis (3a) and cephalometric tracing for surgical planning (3b) were performed. Below, images of the operation with Le Fort I osteotomy in the maxilla (3c), sagittal mandible osteotomy (3d), and bichectomy (3e).

Phase 2 - Bimaxillary orthognathic surgery, lip filling with hyaluronic acid, and bichectomy at the same surgical time, aided by the Nemoceph™ software for surgical planning (Fig. 3). After orthognathic surgery, the patient was instructed to remain at rest for 15 days, leaving only to attend the postoperative return visits once a week for six weeks. The patient was also instructed not to make any physical effort or to expose herself to the sun for 30 days, and to maintain a pasty diet for 40 days.

**Fig. 4:**
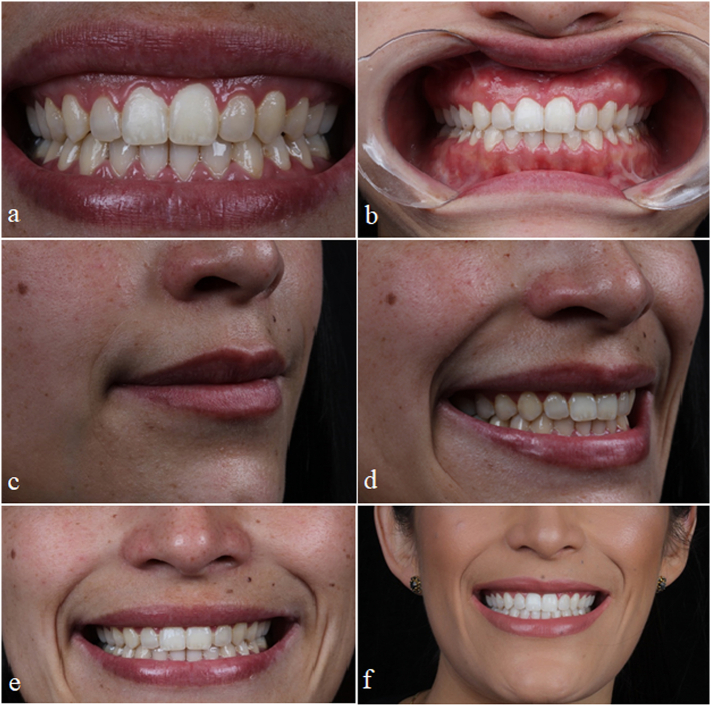
Follow-up 60 days after orthognathic surgery (4a) and gingival plastic surgery in teeth 11, 12, 13, 22, and 23 (4b). Filling of the upper lip with hyaluronic acid (4c and 4d). Initial photo before (4e) and after (4f) tooth bleaching.

**Fig. 5:**
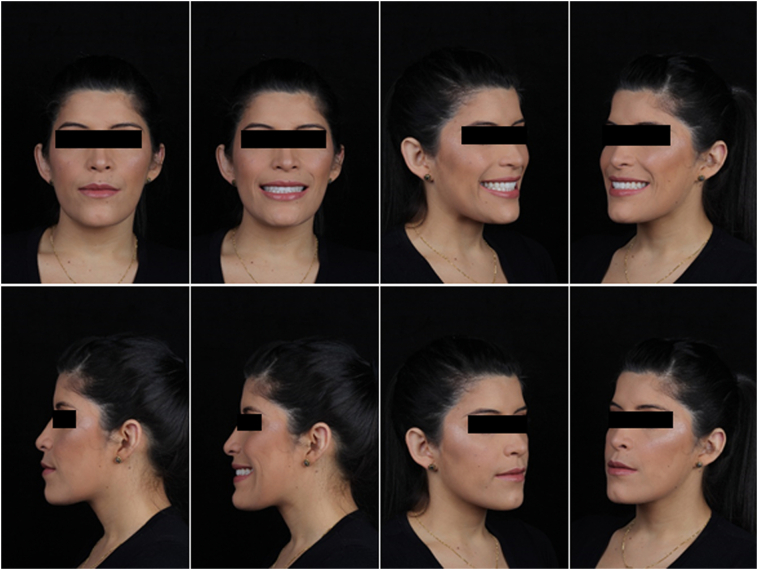
Final photos of the case.

Phase 3 - Integrated dentolabial harmonization process and gingival plastic surgery with surgical laser 60 days after removing the orthodontic appliance and in-office tooth bleaching (Figs. 4 and 5).

This case was conducted by an orthodontist and by the Oral and Maxillofacial Surgery and Traumatology team of the dentist staff at São Vicente de Paulo Hospital. Throughout the process, the patient demonstrated good adherence and tolerability to treatment.

## 3 Clinical discussion

The expected benefits of orthognathic surgery range from optimizing physiological functions to improving the functions involved in social interactions [6]. In fact, esthetics represents one of the main motivations of the patient for surgery [6], and also minimally invasive cosmetic treatments have been increasingly after, with the aim of reshaping facial proportions and symmetry [3]. In this sense, the literature meets the objective of the present case report, which was to demonstrate the use of minimally invasive techniques associated with orthognathic surgery and orthodontics, a strong tendency in a current maxillofacial rehabilitation.

In fact, orthognathic surgery alone has already shown excellent results in terms of improving overall patient satisfaction. Aiming to assess oral health-related quality of life before and after orthognathic surgery, Rezaei et al. [7] examined 112 adult patients with class III skeletal malocclusion using the Short Form Oral Health Impact Profile (OHIP-14) and Orthognathic Quality of Life (OQLQ) questionnaires. Their results showed that orthognathic surgery improved the quality of life of these patients, as well as their satisfaction, self-confidence, and oral function. Such findings corroborate those of the present case report, considering the patient was extremely satisfied with the functional and esthetic findings obtained after the multidisciplinary treatment.

Orthognathic surgery often involves orthodontic treatment before and after surgical skeletal repositioning, and this three-stage sequence is the most popular method due to the greater likelihood of achieving occlusal stability after surgery [8]. However, an alternative method called “Surgery First” involving skeletal repositioning before the orthodontic treatment has been described more recently, showing potential advantages in reducing treatment time and improving facial esthetics at the beginning of the treatment [9].

Pelo et al. [10] conducted a study to evaluate the differences perceived by patients of the “traditional orthognathic approach” and “surgery first.” The post-surgical facial and masticatory improvement led to a better quality of life in both groups. However, the traditional approach group showed greater initial frustration related to the progressive worsening of the facial profile and masticatory function due to initial dental decompensation. The level of patient satisfaction may decrease before surgery due to the lack of knowledge of patients about the phases of orthodontic treatment. Thus, as occurred in the present case, it is up to the multidisciplinary team to raise patient awareness about the therapeutic phases to potentially increase the level of satisfaction with the treatmen [7].

Minimally invasive cosmetic improvements have been increasingly sought for reshaping facial proportions and increasing symmetry [3]. In the search for ideal esthetic results, it is essential that specialists have knowledge of facial anatomy, and treatment should be focused on the needs of the patient so that a specific esthetic approach for each case is properly selected [11]. The planning and execution of the treatment at the present case involved a stage of tooth-lip harmonization with the application of HA in order to improve the patient's esthetic results. Basically, this material is injected subcutaneously to add volume, change the conformation of the surface, thicken the skin or tissues or fill a rhytid. All of these points are a form of sculpture, which results in a significant change in facial appearance [4].

Wollina & Goldman [12] described the case of a 17-year-old patient subjected to orthognathic surgery 4 years earlier. Due to this procedure, the nasolabial angle was wide and the upper lip vermilion was thin and seemed flat. The patient received 0.6 mL of HA filling to volumize the upper and lower lips, obtaining an improvement in the quality of life and self-esteem. Thus, despite the various advantages offered by orthognathic surgery, unwanted esthetic changes may occur, which can be corrected by minimally invasive techniques.

As a multidisciplinary approach is often necessary to achieve esthetic harmony between face, teeth, lips and gums, interaction with various specialties of Dentistry must be used to solve the complexity of each patient's case [13]. Considering that a harmonious smile is influenced by several factors such as minimal gingival exposure, presence of healthy gingival tissue in interproximal spaces, symmetry between the upper gingival margin and upper lip, harmony between anterior and posterior segments, and correct proportions and anatomy [14], this case also involved a stage of gingival repair and tooth whitening, important steps in the search for an excellent result.

## 4 Conclusion

Currently, a closer look at Dentistry has been expanding and modifying rehabilitation standards, with the integration of several disciplines, new multidisciplinarity concepts, and more comprehensive views offering possibilities for additional procedures that may help to restore function, health, and esthetics of patients. The present case report reaffirms the benefits of orthognathic surgery associated with minimally invasive facial harmonization procedures used to refine the technique. The surgical procedure not only corrected the functional complaint of the patient but also played an important role in improving facial harmony, contributing significantly to self-esteem.

## Consent and ethics

Written informed consent was obtained from the patient for publication of this case report and accompanying images. A copy of the written consent is available for review by the Editor-in-Chief of this journal on request.

## Sources of funding

None.

## CRediT authorship contribution statement

All authors actively contributed to the realization of this work, as detailed: Alessandra Kuhn-Dall'Magro: Conceptualization, methodology, supervision; Letícia Copatti Dogenski: Investigation, writing - original draft, writing - review & editing; Eduardo Dall'Magro: Writing - review & editing, visualization; Nicoly Schmidt Figur: Investigation, writing - original draft; Micheline Sandini Trentin: Writing - review & editing, visualization; João Paulo De Carli: Writing - review & editing, visualization.

## Guarantor

Alessandra Kuhn-Dall'Magro.

## Research registration number

Not applicable.

## Declaration of competing interest

None.
